# COHESION: core outcomes in neonatal encephalopathy (protocol)

**DOI:** 10.1186/s13063-021-05030-0

**Published:** 2021-02-08

**Authors:** Fiona A. Quirke, Patricia Healy, Elaine Ní Bhraonáin, Mandy Daly, Linda Biesty, Tim Hurley, Karen Walker, Shireen Meher, David M. Haas, Frank H. Bloomfield, Jamie J. Kirkham, Eleanor J. Molloy, Declan Devane

**Affiliations:** 1 Health Research Board Neonatal Encephalopathy PhD Training Network (NEPTuNE), Galway, Ireland; 2 Health Research Board – Trials Methodology Research Network (HRB-TMRN), Galway, Ireland; 3 College of Medicine, Nursing and Health Sciences, National University of Ireland Galway, Galway, Ireland; 4 School of Nursing and Midwifery, National University of Ireland Galway, Galway, Ireland; 5 Family Support Liaison, Irish Neonatal Health Alliance, Wicklow, Ireland; 6 Advocacy and Policymaking, Irish Neonatal Health Alliance, Wicklow, Ireland; 7 Qualitative Research in Trials Centre (QUESTS), National University of Ireland Galway, Galway, Ireland; 8 Paediatrics and Child Health, Trinity College Dublin, Dublin, Ireland; 9 RPA Newborn Care, Sydney Local Health District, Sydney, Australia; 10 Birmingham Women’s and Children’s NHS Foundation Trust, Birmingham, UK; 11 Department of Obstetrics and Gynaecology, Indiana University School of Medicine, Indianapolis, USA; 12 Liggins Institute, University of Auckland, Auckland, New Zealand; 13 Centre for Biostatistics, University of Manchester, Manchester, UK; 14 Department of Neonatology, Children’s Hospital Ireland at Crumlin and Tallaght, Coombe Women and Infants University Hospital, Dublin, Ireland; 15 Evidence Synthesis Ireland, National University of Ireland Galway, Galway, Ireland; 16 Cochrane Ireland, National University of Ireland Galway, Galway, Ireland

**Keywords:** Core outcome set, High-income countries, Low- to middle-income countries, Delphi, Real-time Delphi, PPI, Neonatal encephalopathy, Outcomes

## Abstract

**Background:**

Neonatal encephalopathy is a complex syndrome in infants that predominantly affects the brain and other organs. The leading cause is a lack of oxygen in the blood reaching the brain. Neonatal encephalopathy can result in mortality or complications later in life, including seizures, movement disorders and cerebral palsy. Treatment options for neonatal encephalopathy are limited mainly to therapeutic hypothermia, although other potential treatments are emerging. However, evaluations of the effectiveness of treatments are challenging because of heterogeneity and inconsistency in outcomes measured and reported between trials. In this paper, we detail how we will develop a core outcome set to standardise outcomes measured and reported upon for interventions for the treatment of neonatal encephalopathy.

**Methods:**

We will systematically review the literature to identify outcomes reported previously in randomised trials and systematic reviews of randomised trials. We will identify outcomes important to parents or caregivers of infants diagnosed with and who have received treatment for neonatal encephalopathy. We will do this by conducting in person or by video teleconferencing interviews with parents or caregivers in high-income and low- to middle-income countries. Stakeholders with expertise in neonatal encephalopathy (parents/caregivers, healthcare providers and researchers) will rate the importance of identified outcomes in an online Delphi survey using either a three-round Delphi survey or a “Real-Time” Delphi survey to which stakeholders will be allocated at random. Consensus meetings will take place by video conference to allow for an international group of stakeholder representatives to discuss and vote on the outcomes to include in the final core outcome set (COS).

**Discussion:**

More research is needed on treatments for neonatal encephalopathy. Standardising outcomes measured and reported in evaluations of the effectiveness of interventions for the treatment of neonatal encephalopathy will improve evidence synthesis and improve results reported in systematic reviews and meta-analysis in this area. Overall, this COS will allow for improved treatments to be identified, heterogeneity in research to be reduced, and overall patient care to be enhanced.

**Trial registration:**

This study is registered in the Core Outcome Measures for Effectiveness (COMET) database http://www.comet-initiative.org/Studies/Details/1270.

## Background

Neonatal encephalopathy is an umbrella term encompassing a complex neurological syndrome in infants born at 35 weeks of gestation or later (as reported by the American College of Obstetricians and Gynecologists’ Task Force on Neonatal Encephalopathy [1]). Lee et al. [2] estimated that, in 2010, approximately 1.15 million infants globally had developed neonatal encephalopathy associated with intrapartum events, of which 96% were born in low- and middle-income countries. It was also estimated that around 287,000 babies with neonatal encephalopathy died in 2010, while 414,000 survived with varying neurodevelopmental impairments [2].

Neonatal encephalopathy is associated in the early days after birth with seizures, an altered state of consciousness, depression of tone and reflexes and difficulty in maintaining normal respiration [3]. Neonatal encephalopathy is also associated with early mortality in the newborn and with long-term morbidity, including poor neurodevelopmental outcomes [4–6]. Neonatal encephalopathy is associated with a range of maternal risk factors including hypertension and pre-eclampsia, hypothyroidism [7], foetal growth restriction [8], diabetes [9], prolonged labour [10], bleeding in pregnancy, hypoxia, acute intrapartum events [8] and placental complications [11, 12]. Foetal and neonatal risk factors include hypoxic ischaemia, giving rise to hypoxic ischaemic encephalopathy [8, 13], systemic infection, intracranial infections (viral or bacterial) [14], metabolic disorders (including mitochondrial disorders and organic acidaemias) [15], neonatal stroke [16], genetic and epigenetic risk factors [17], intracranial haemorrhage, epileptic syndromes and neurodegenerative disorders [16] among others [3].

Hypoxic ischaemic encephalopathy (HIE) is a subgroup of neonatal encephalopathy [18] that develops primarily as a result of hypoxic ischaemia in the newborn. However, hypoxic ischaemia may be present in some form as a secondary event in infants with neonatal encephalopathy [18]. Hypoxia refers to diminished oxygen in the tissues secondary to asphyxia (altered gas exchange), and ischaemia refers to a deficiency in the flow of blood available for perfusion. Both can cause profound neurological damage when brain cells are affected. Badawi et al. [8] estimate that hypoxic ischaemia (asphyxia) is a contributing factor in approximately 29% of neonatal encephalopathy cases and the leading factor in a further 4% of cases.

The impact on parents caring for an infant with neonatal encephalopathy was investigated by Lemmon et al. [19], as part of a longitudinal cohort study from 2011 to 2014. Twenty interviews were conducted with parents of infants who received therapeutic hypothermia. These parents described a sense of loss (i.e. due to complications in the perinatal period, disrupted bonding with the infant, etc.), a disrupted way of life (i.e. job loss, time given to attend medical appointments, etc.) and a sense of loss of how they perceived their life should have been. Families also described feeling a sense of guilt or regret surrounding the balance of maternal health decisions, infant treatment, wider familial needs/ priorities, and financial responsibilities. Many parents also described how their role as a parent had evolved into that of an advocate for their child. Interventions for the treatment of neonatal encephalopathy vary depending on the underlying cause of encephalopathy. Therapeutic hypothermia for the treatment of neonates with moderate to severe HIE improves prognosis. A Cochrane systematic review including 11 randomised controlled trials with 1505 late preterm (35 to 37 weeks) and term infants with moderate to severe encephalopathy demonstrated that therapeutic hypothermia reduced death and neurodevelopmental disability in survivors by 25% [20, 21]. It is, therefore, now used as the standard treatment for moderate to severe HIE in many countries worldwide [22, 23]. However, its narrow therapeutic window means it needs to be initiated within the first 6 h after hypoxic ischaemia to optimise therapeutic benefit. Therefore, an early diagnosis is crucial for this therapy to be most efficient [24] and additional treatment options are needed.

In addition to therapeutic hypothermia, other agents are being investigated for their neuroprotective properties as either an adjuvant to therapeutic hypothermia and other treatments, or as a stand-alone therapy. Melatonin and erythropoietin are both emerging as potential adjuvant therapies to therapeutic hypothermia to improve neurodevelopmental outcomes in newborns with neonatal encephalopathy [25–28]. A study by El Farargy and Soliman [29] showed that magnesium sulphate (M_g_SO_4_) administered in combination with melatonin showed a positive effect in reducing brain injury when administered to infants diagnosed with HIE.

However, a significant difficulty in evaluating the effectiveness of new therapies to improve neurodevelopmental outcomes and overall neonatal health is the lack of standardisation of the outcomes reported in these trials. Heterogeneity in outcomes measured and reported frequently hinders comparing and contrasting findings across multiple studies. It also leads to waste in research when findings cannot be used to inform the best care for patients [30, 31]. For example, Jacobs et al. [21] conducted a Cochrane systematic review of 11 trials (1505 infants) evaluating the effectiveness of therapeutic hypothermia in encephalopathic asphyxiated newborn infants but were unable to analyse several a priori secondary outcomes because these were not reported in the included trials. Likewise, in a review by Ruegger et al. [32] investigating xenon as an adjuvant to therapeutic hypothermia, they judged the risk of attrition bias in the primary outcome as “unclear” due to incomplete outcome reporting. A lack of standardisation in trials limits the ability of researchers and healthcare providers to improve patient treatment and care as any actual benefits or harms of the therapy are not clear due to the substantial differences in outcomes between trials.

One way to address this problem is to develop and apply agreed standardised sets of outcomes, known as “core outcome sets” (COS) [31]. A COS represents the minimum set of outcomes to be measured and reported in all trials, and other studies, on a specific condition, while accepting that if outcomes outside of the COS are also important in the context of the individual study they should also be measured for that study [31]. This use of the COS as a minimum set of outcomes to be measured and reported across an entire research area would allow for the results of trials and other studies to be effectively compared, contrasted and combined, as appropriate. However, the successful uptake of a COS will depend ultimately on overcoming certain barriers including involvement of relevant stakeholders, a well-structured consensus process for prioritising outcomes and a plan for dissemination of the COS when complete [33].

The COHESION study will develop a COS for use in randomised trials, and other studies, for evaluating the effectiveness of interventions for the treatment of neonatal encephalopathy.

## Methods

The development of this COS will adhere to recommendations set out by the COMET initiative [31]. In the development of this protocol, we adhere to the Core Outcome Set-STAndards for Development (COS-STAD) recommendation [34], and follow the COS-STAP statement (Core Outcome Set-STAndardised Protocol Items) [35] (See Appendix A), for developing a COS protocol.

**Fig. 1:**
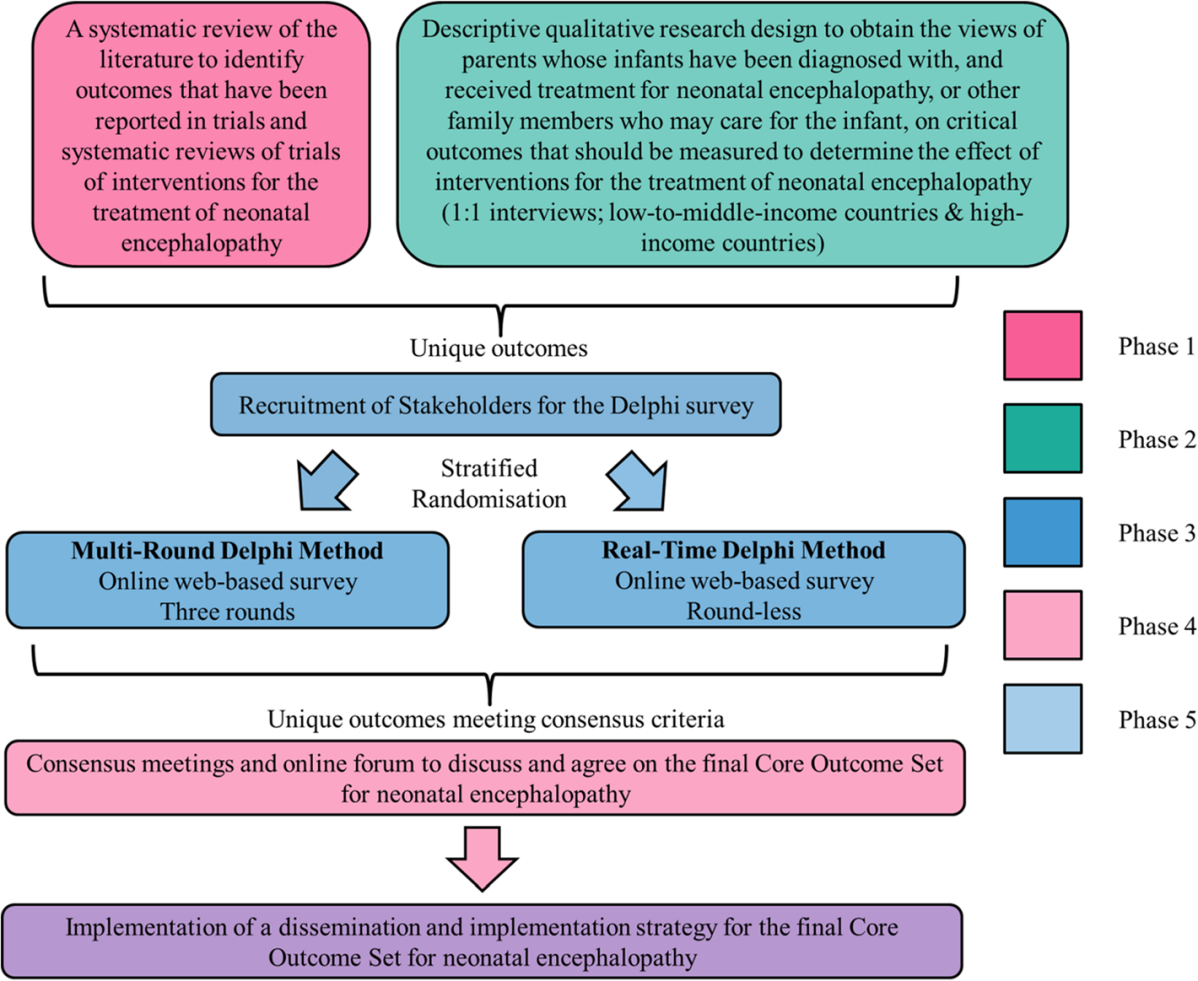
Schematic of COS development

We will develop the COS through five discrete, yet complementary phases (see Fig. 1):

- Phase 1: A systematic review of the literature to identify outcomes that have been reported in randomised trials and systematic reviews of randomised trials of interventions for the treatment of neonatal encephalopathy;
- Phase 2: A qualitative component using interviews to obtain the views of parents whose infants have been diagnosed with, and received treatment for, neonatal encephalopathy or other family members who may care for the infant, on critical outcomes they feel should be measured to determine the effect of treatment(s) for neonatal encephalopathy;
- Phase 3: Development of a preliminary COS (informed by Phases 1 & 2) through stratified randomisation of key stakeholders to one of two web-based, Delphi surveys;
- Phase 4: Consensus meeting to discuss and agree on the final neonatal encephalopathy COS;
- Phase 5: Dissemination and Implementation strategy for the final COS.

The steering group for COHESION consists of neonatologists, obstetricians, midwives, a neonatal nurse practitioner, parents (i.e. public and patient involvement (PPI) representatives), experts in COS development and researchers with expertise in neonatal encephalopathy. The collective knowledge of this group will inform the development of this COS.

### Phase 1: Systematic review

#### Research question: what are the outcomes reported in studies for the treatment of neonatal encephalopathy?

We will carry out a systematic review of randomised trials and systematic reviews of randomised trials evaluating the effectiveness of interventions for the treatment of neonatal encephalopathy to identify and collate reported outcomes.

#### Inclusion criteria

##### Types of studies

The types of studies are randomised trials and systematic reviews of randomised trials (with and without meta-analyses) evaluating the effectiveness of interventions for the treatment of neonatal encephalopathy.

##### Types of participants

Patients will include infants treated for neonatal encephalopathy or HIE, greater than or equal to 35 weeks gestation. Where there is a mixed gestational age reported, at least 80% of infants must be greater than, or equal to, 35 weeks gestation.

##### Types of interventions

The types of interventions are any intervention used for the treatment of neonatal encephalopathy or HIE. Comparison(s) include any comparator intervention(s) for the treatment of neonatal encephalopathy. This may involve an alternative treatment, standard care, a placebo treatment or no treatment.

##### Types of outcomes

All of the outcomes reported in the included studies will be recorded, along with the timing of the outcomes. 

##### Search methodology

We will perform two separate searches for:

- Randomised Controlled Trials, through
  - CENTRAL
  - MEDLINE and Embase
- Systematic Reviews of Randomised Controlled Trials, through
  - Cochrane Database of Systematic Reviews (CDSR)
  - MEDLINE and Embase

In addition, the World Health Organization’s International Clinical Trials Registry Platform (WHO-ICTRP) will also be searched for ongoing trials.

##### Assessment for eligibility

The titles and abstracts of citations identified from our search will be screened and reviewed independently by two reviewers (FAQ and DD or PH). Full texts of potentially relevant studies will be assessed to determine their eligibility. If there is uncertainty among the reviewers regarding the inclusion of a study or its relevance at the screening stage, a third reviewer (DD or PH) will be consulted.

##### Data extraction

Data will be extracted from each study on study design, author details, year and journal of publication, the country in which the study was conducted, targeted condition, criteria for the diagnosis of neonatal encephalopathy, interventions under investigation and all outcomes as they are reported in the studies (including measurement time-points). Extracting this data ensures the inclusion criteria are met for included studies. Extracting the country in which the study was conducted will allow us to assess any potential differences in outcomes between high- and low- to middle-income countries that may emerge from the interviews with parents. One review author (FAQ) will extract data while a second reviewer (DD or PH) will complete an analysis and verification check of 50% of the outcomes extracted. If there are discrepancies found, all extracted outcomes will be verified by the second reviewer (DD or PH). Disagreement will be resolved through discussion with a third reviewer (DD or PH).

##### Data analysis and presentation

Data will be tabulated using Excel so that each study is listed, and all outcomes measured in each study are displayed separately. Outcomes identified from the systematic review will be reviewed by the COHESION Steering Group, which includes PPI representatives. Outcomes will be grouped under major domains (e.g., maternal outcomes, neonatal, infant, child and adult outcomes, and others, e.g. health economic consequences), as pre-determined by the Steering Group. Domains will then be reviewed by the Steering Group to discuss where gaps are in important domains.

### Phase 2: Qualitative exploration of outcomes important to parents/caregivers whose infants have been diagnosed with, and received treatment for, neonatal encephalopathy

#### Research question

*What are the outcomes regarded as potentially important in neonates diagnosed with, and treated for, neonatal encephalopathy from the perspective and experiences of parents or other family members who may care for the infant?*

Systematic reviews are more likely to highlight outcomes identified as important by researchers [31]. We will carry out interviews to obtain the views of mothers, their partners and other family members who may care for the infant, on critical outcomes that they feel should be measured to determine the effect of interventions for the treatment of neonatal encephalopathy.

#### Design

This phase will be informed by descriptive qualitative research design, using interviews, to obtain the perspectives of parents or other family members who may care for the infant, on outcomes they judge important for inclusion in the Delphi survey in Phase 3. Descriptive qualitative research allows the perspective of those experiencing the phenomenon (in healthcare research, this often directly relates to the experiences of a patient) to be captured [36, 37]. As we cannot conduct interviews with the patients (neonates) in this study, we will use qualitative research to elicit the perspective of women, their partners, and other family members who may care for the infant, on outcomes they judge important. We will report this qualitative phase following reporting recommendations for qualitative research methods in COS development as outlined by Jones et al. [38] (Table 1).

**Table 1:** Reporting recommendations for qualitative research methods in COS development, as developed by Jones et al. [38]

**1**	Research aims and relationship with broader COS development process
**2**	Sampling approach
**3**	Type of data collection methods (e.g. interviews, focus groups, combination); content and derivation/ justification (e.g. topic guide)
**4**	Analytical approach and justification
**5**	Sample characteristics and participants numbers
**6**	Findings related to outcome domains (concordant with research aims)
**7**	Report approaches to ensuring rigour (e.g. multiple perspectives on the data, respondent validation) and consider reflexive content
**8**	Discuss the strengths and limitations of the approach

#### Sampling

*Where?*

We will recruit stakeholders for one-to-one interviews from high-income countries (Ireland, Australia, United Kingdom and United States) and low- to middle-income countries (Kenya, India and Pakistan).

*Who?*

Potential participants will be invited to join this study. The participants are parents whose infants have been diagnosed with and received treatment for neonatal encephalopathy, or other family members who may care for the infant from each country (i.e. caregivers). Infants will have been born at 35 weeks gestation or later, with a birth weight considered healthy for each location. In low- to middle-income countries, a diagnosis of birth asphyxia and/or neonatal encephalopathy will be accepted. If it is not possible to recruit enough parents or caregivers of infants who have been diagnosed with and received treatment for birth asphyxia or neonatal encephalopathy, we will try to recruit parents of infants with neonatal encephalopathy who did not receive treatment. We will recruit a minimum of five parents per country initially, but final participant numbers will be determined by data saturation, i.e. the point in which no additional outcomes are being suggested [39].

*How?*

Steering group members will identify Gatekeepers in each respective country. Gatekeepers will consist of researchers, healthcare professionals working in the field of neonatal encephalopathy, and PPI representatives from voluntary organisations supporting parents of children with neonatal encephalopathy. The Gatekeepers will act to identify potential participants through their local knowledge in their particular country. A purposive sampling strategy will be employed in which the Gatekeeper will identify participants through any one of the following means: professional organisations, advocacy groups, parent support groups, previous participation in related research (and where consent permits contact as proposed).

#### Interview format

One-to-one interviews will be the primary method of data collection. Interviews have been used in obtaining patient/user-important outcomes previously [40–42]. The benefit of one-to-one interviews is that it allows study participants who may not speak freely in a group setting to participate and share their views without the influence of other people’s opinions.

The one-to-one interviews will focus on obtaining the views of parents/caregivers whose infants have been diagnosed with and treated for neonatal encephalopathy (or birth asphyxia), on critical outcomes that should be measured to determine the effect of interventions for the treatment of neonatal encephalopathy. The interviews will follow a semi-structured format, where participants will initially be prompted by open questions to encourage discussion. PPI representatives on the COHESION steering group will give guidance on developing and reviewing the interview guide. The interview guide may develop iteratively during the interview process.

##### High-income countries

As English is spoken widely in both of these countries, one-to-one interviews will be offered through video conferencing, which will be available through any computer or handheld device including mobile phones. Once the participants have been identified, they will be sent an information pack via email (outlining the purpose, expectations of participants, benefits and harms to participation in this study, how data will be used, right to withdraw, voluntary participation and an offer to answer any questions). Potential participants will be invited to respond directly to the lead researcher (FAQ) if they want to participate. Once consent is received from the participant to take part in the interview, these interviews will be carried out by Steering Group members (FAQ and LB).

##### Low- to middle-income countries

The Gatekeepers will recruit local researchers with experience in interviewing to conduct the interviews. We have considered the need to ensure that potential participants are given sufficient information on which to base a decision on whether or not they would like to participate in the interview. We cannot be confident that potential participants, in low- to middle-income countries, in particular, will have the literacy levels necessary to read and understand a participant information leaflet and consent form. For example, approx. 40% of the Kenyan population are illiterate but regional literacy varies widely from, for example, approx. 87% in Nairobi to 8% in North Eastern Province (see: https://www.eldis.org/document/A31868). Interviewers (local researcher or healthcare professional) will speak both English and the local dialect of participants fluently. The interviewer will pre-record a verbal explanation of the contents of the information pack (i.e. the purpose of the project, expectations of participants, benefits and harms to participation in this study, how data will be used, right to withdraw, voluntary participation and an offer to answer any questions which may also be directed to the lead researcher (FAQ) to answer) for participants to listen to if they have low literacy levels. Alternatively, they can read the Participant Information Leaflet themselves. Participants who agree to participate will sign (either through signing their signature or an “X”) on the consent form indicating that they have given their consent to take part. A copy of the signed consent form will be sent to the COHESION research team at the earliest opportunity. The interviewer will conduct the interviews in the native language using a semi-structured interview schedule. The Participant Information Leaflet, Consent Form and Interview Guide will be forward- and back-translated and compared against the original English-language document and any discrepancies will be resolved before the interviews commence. The interview will be audio-recorded, and the recording will be transcribed. Transcripts will be translated into English by the interviewer and then returned to the COHESION research team.

#### Data analysis

Using the principles of thematic analysis, the interview data will be coded to describe the content in terms of outcomes; patterns will then be established across the codes to identify the themes noted by the participants as important measures to determine the effect of treatment(s) for neonatal encephalopathy. The context of why the outcome was important to the participant will also be identified and noted. This will allow for clarification of the importance of the outcomes in subsequent phases of the COS development such as the development of plain-language summaries of outcomes for the Delphi surveys and justification of outcomes going forward to the consensus meeting(s). As highlighted by Williamson et al. [43] and Keeley et al. [44], qualitative interviews are also beneficial for improving the language used to describe outcomes for the Delphi survey and also allow for the scope of the outcomes as described by parents, to be better captured.

### Phase 3: Delphi surveys

Consensus must be reached among stakeholders on the final outcomes included in the COS. In COS development, the use of Delphi surveys, in combination with other consensus methods such as focus groups or interviews among other techniques, or as the primary method of consensus building, is common [45].

#### Participants

We will recruit stakeholders with expertise in neonatal encephalopathy to participate in our Delphi survey. Stakeholders will be identified through email invitations, electronic discussion lists, individuals who have contributed to work or research in this area previously, other experts in the field of neonatal encephalopathy who have publications in this field as identified through carrying out the systematic review and qualitative interviews, through international professional organisations, and support networks. Stakeholders will be grouped into three broad groups: (a) parents/caregivers of infants who have been diagnosed with, and received treatment for, neonatal encephalopathy, (b) healthcare providers, including policymakers and (c) researchers with expertise in the area of neonatal encephalopathy.

#### Methods

In this part of the COS development process, we are incorporating a randomised trial to identify whether different outcomes are prioritised when using a Real-Time Delphi method compared with a Multi-Round Delphi method in the development of a COS on interventions for the treatment of neonatal encephalopathy. Participants will be randomised to participate in one of two Delphi surveys. One survey will involve a three-round approach, while the other involves a single-round, Real-Time Delphi survey. In both surveys, stakeholders will be asked to rate the importance of the same list of outcomes that emerge from the interviews with parents/caregivers and the systematic review. In both surveys, participants will be asked to rate the importance of each outcome on a 9-point Likert scale (i.e. 1–3 limited importance, 4–6 important but not critical and 7–9 critical) [31]. In the Multi-Round Delphi, feedback will be given to participants at the end of each round. Participants will be given the opportunity to change how they rated outcomes in rounds 2 and 3 based on this feedback. Participants who have taken part in the first round of the survey will be invited to participate in the second round. Likewise, those who have participated in the second round will be invited to participate in the final round of the survey. In the single-round “Real-Time” Delphi, feedback will be available to participants in 'real-time' when they enter the survey. This will give the participants the opportunity to review the outcomes and modify how they rate the outcome if they wish. They will also be reminded by email to re-visit and re-rate outcomes before the survey ends. The feedback given in both surveys will consist of the individual respondents’ rating and the proportion of people scoring each point in the 9-point Likert scale for each outcome, for (a) each stakeholder group (i.e. parents/caregivers; healthcare providers, including policy makers, and researchers with expertise in the area of neonatal encephalopathy) and, (b) overall, across all groups as defined by Table 2. All unique outcomes that emerge at the end of both survey arms will be combined and brought forward to the consensus meeting(s). For more information on the method used in this trial, please see the *In-press* protocol that accompanies this paper “*Multi-round compared to Real-Time Delphi for consensus in Core Outcome Set (COS) development: A randomised trial”.*

**Table 2:** Consensus classification

Consensus classification	Description	Definition
Consensus in (parent-weighted vote)	Consensus that outcome should be included in the core outcome set	70% or more participants overall scoring as 7 to 9 AND < 15% participants scoring a 1 to 3 OR > 70% or more of parent group scoring as 7 to 9
Consensus out	Consensus that outcome should not be included in the COS	50% or fewer participants scoring as 7 to 9 in each stakeholder group
Neither consensus in nor consensus out (undetermined consensus)	Uncertainty about importance of outcome so retain for next round	Anything else

### Phase 4: Consensus meeting(s)

#### Objective

The objective of the consensus meeting is to achieve agreement on the final COS through an online meeting of international key stakeholders with expertise in neonatal encephalopathy.

#### Participants

Stakeholders in the consensus meeting(s) will include at least two representatives of parents or caregivers whose infants have been diagnosed with or treated for neonatal encephalopathy, healthcare providers and researchers/academics with expertise in neonatal encephalopathy treatment. We aim to have a global spread of stakeholders participating in the consensus meeting(s). This will be accommodated by facilitating multiple meetings via “Zoom” teleconference.

#### Outcomes

The outcomes emerging from the Multi-Round Delphi survey and the Real-Time Delphi survey will be pooled and duplicates removed. This combined list of unique outcomes will be put forward to the consensus meeting(s). Outcomes voted as “consensus out” will be presented as an independent set and stakeholders will be asked if they accept the omission of these outcomes. Outcomes voted as “consensus in” (Table 2) will then be presented independently and discussed individually, and stakeholders will be asked to vote on the inclusion of these outcomes in the COS. Any outcomes that were voted neither “consensus in” nor “consensus out” will be discussed, and the stakeholders will vote if any of these outcomes should be included.

#### Schedule

Materials will be distributed in advance of the meeting(s) to inform discussions. A non-voting facilitator will ensure that each meeting is (i) collaborative; (ii) cooperative and non-competitive; (iii) egalitarian, providing equal input from all participants; (iv) inclusive with all participants contributing to discussions and (v) participatory.

As highlighted by Gargon et al. [46], there are often difficulties in having an international representation at the consensus meeting to determine the final COS. Some of the obstacles mentioned in having an international consensus meeting include financial and human resources [46]. We propose to conduct the consensus meeting for COHESION via video conference. To optimise international representation to decide on the final COS, multiple meetings may need to be held, with corresponding time zones taken into account. The unique outcomes from these consensus meeting(s) will be pooled and used to populate an online discussion forum. Using this online forum, stakeholders who attended the consensus meeting(s) can discuss outcomes and cast their final vote for an outcome to be included in the final COS. The outcomes emerging from this online voting will make up the final COS for neonatal encephalopathy.

### Phase 5: Dissemination and implementation strategy

The dissemination and implementation strategy for COHESION is guided by the Health Research Board (HRB, Ireland), knowledge transfer strategy: (i) Monitor; (ii) Inform; (iii) Knowledge Exchange; (iv) Persuade; (v) Network and (vi) Support. Our proposed methods of dissemination and implementation include, but not limited to:

- Targeting key stakeholders with interest in neonatal encephalopathy such as survey participants, health care providers in maternity hospitals, neonatal encephalopathy and maternity care researchers, Colleges of Physicians, Colleges of Obstetrics and Gynaecologists’, national and international societies and organisations for circulating to their members such as:
  - The Cochrane Pregnancy and Childbirth Group
  - CROWN (Core Outcomes in Women’s and Newborn Health) Initiative
  - Society for Reproductive Investigation (SRI)
  - Perinatal Society of Australia and New Zealand (PSANZ)
  - British Association of Perinatal Medicine (BAPM)
  - Paediatric Academic Society (PAS)
  - Paediatric Research Society (PRS)
  - European Society for Paediatric Research (ESPR)
  - International Confederation of Midwives (ICM)
  - International Federation of Gynecology and Obstetrics (FIGO)
  - Irish Paediatric Association (IPA)
  - Irish Neonatal Health Alliance (INHA)
  - Global Alliance for Newborn Care (GLANCE)
  - Miracle Babies Foundation (MBF)
  - Life’s Little Treasure Foundation (LLTF)
  - Council of International Neonatal Nurses (COINN)
  - European Foundation for the Care of Newborn Infants (EFONI)
  - Union of European Neonatal & Perinatal Societies (UENPS)
  - Hope For HIE
- Establishing networks and collaborations with an interest in COS development (COMET (Core Outcome Measures in Effectiveness Trials) team for inclusion in the database of COS (http://www.comet-initiative.org/).
- Engagement with research funders, national insurers, guideline developers, trial registries and guideline developers.

Ways in which will achieve dissemination to the target audience include open access publications, presentations at relevant conferences, website updates (http://nbci.ie/about-neptune/), posters, events, research briefs, newsletters, press release, podcasts and social media updates.

## Discussion

Interventions for the treatment of neonatal encephalopathy vary depending on the underlying cause of encephalopathy. Therapeutic hypothermia has become the most common treatment for neonates with neonatal encephalopathy caused by hypoxic ischaemia. Therapeutic hypothermia is not without its limitations. As mentioned, it is a time-limited treatment most beneficial when initiated before 6 h, and its optimal way to provide cooling is uncertain [21]. Other interventions are emerging as adjuvant treatments with therapeutic hypothermia or as stand-alone treatments. As these new treatment interventions are evaluated in trials, it is important that the same core outcomes are used to determine the effect of the treatment. This will help minimise heterogeneity and provide greater opportunity for synthesising evidence from different trials.

Currently, there is no published COS for studies evaluating the effects of interventions for the treatment of neonatal encephalopathy. The development of this COS will ensure there is transparency surrounding the collection and reporting of a minimum dataset that is agreed by key stakeholder consensus. This should help to reduce inconsistencies and heterogeneity in outcomes reported in trials involving neonatal encephalopathy treatment.

## Data Availability

All datasets used and/or analysed during the COHESION study will be held by COHESION team as f.quirke1@nuigalway.ie.

## Appendix

**Table 3:** Core outcome set-STAndardised protocol items

TITLE/ABSTRACT			
Title	1a	Identify in the title that the paper describes the protocol for the planned development of a COS	Page 0
Abstract	1b	Provide a structured abstract	Page 0–1
INTRODUCTION			
Background and objectives	2a	Describe the background and explain the rationale for developing the COS, and identify the reasons why a COS is needed and the potential barriers to its implementation	Pages 2–7
2b	Describe the specific objectives with reference to developing a COS		Pages 6–7
Scope	3a	Describe the health condition(s) and population(s) that will be covered by the COS	Pages 2–5
3b	Describe the intervention(s) that will be covered by the COS		Pages 2–7
3c	Describe the context of use for which the COS is to be applied		Pages 6–7
METHODS			
Stakeholders	4	Describe the stakeholder groups to be involved in the COS development process, the nature of and rationale for their involvement and also how the individuals will be identified; this should cover involvement both as members of the research team and as participants in the study	Pages 14, 17–20
Information sources	5a	Describe the information sources that will be used to identify the list of outcomes. Outline the methods or reference other protocols/papers	Pages 9–16
5b	Describe how outcomes may be dropped/combined, with reasons		Pages 16–20
Consensus process	6	Describe the plans for how the consensus process will be undertaken	Pages 16–20
Consensus definition	7a	Describe the consensus definition	Page 18
7b	Describe the procedure for determining how outcomes will be added/combined/dropped from consideration during the consensus process		Pages 16–20
ANALYSIS			
Outcome scoring/feedback	8	Describe how outcomes will be scored and summarised, describe how participants will receive feedback during the consensus process	Pages 17–18
Missing data	9	Describe how missing data will be handled during the consensus process	Pages 17–18
ETHICS and DISSEMINATION			
Ethics approval/informed consent	10	Describe any plans for obtaining research ethics committee/institutional review board approval in relation to the consensus process and describe how informed consent will be obtained (if relevant)	Page 26
Dissemination	11	Describe any plans to communicate the results to study participants and COS users, inclusive of methods and timing of dissemination	Pages 19–20
ADMINISTRATIVE INFORMATION			
Funders	12	Describe sources of funding, role of funders	Page 26
Conflicts of interest	13	Describe any potential conflicts of interest within the study team and how they will be managed	Page 26

## Abbreviations

COS: Core outcome set
HIE: Hypoxic ischaemic encephalopathy
COMET: Core Outcome Measures in Effectiveness Trials
PPI: Public and patient involvement
COS-STAP: Core Outcome Set-STAndardised Protocol Items
COS-STAD: Core Outcome Set-STAndards for Development
FAQ: Fiona Anne Quirke
DD: Declan Devane
PH: Patricia Healy
LB: Linda Biesty
CENTRAL: Cochrane Central Register of Controlled Trials
CDSRs: Cochrane Database of Systematic Reviews
WHO-ICTRP: World Health Organization’s International Clinical Trials Registry Platform
